# Solar Panels and Political Attitudes

**DOI:** 10.1177/14789299211044868

**Published:** 2021-09-16

**Authors:** Resul Umit

**Affiliations:** ARENA Centre for European Studies, University of Oslo, Oslo, Norway

**Keywords:** policy feedback, political attitudes, climate policy

## Abstract

In the fight against climate change, renewable energy has been subsidised in many countries. With the costs passed onto consumers, governments are paying those, for example, who instal domestic solar panels on top of their homes and feed electricity back into the system at preferential rates. We know that substantial amounts of income flow into households with solar installations as a result, but we do not know much about the political consequences of these programmes. Similar government programmes are known to have resource and interpretative effects on participants, leading to changes in their attitudes. Drawing on three longitudinal surveys from Germany, United Kingdom, and Switzerland, this article analyses whether installation of these solar panels causes meaningful changes in households’ various political attitudes. Using fixed-effect models as the identification strategy, the article reports null results – solar installations do not seem to generate political attitudes. This is good as well as bad news for actors looking to increase the amount of renewable energy produced through solar installations.

## Introduction

Policies to fight climate change have created a new kind of energy producer – households with solar installations on their roof. In addition to the subsidies available for households to instal solar panels, in many countries, they can also feed electricity back into the system at preferential rates. With the the costs passed onto the energy consumers, solar installations are essentially redistributive mechanisms. Indeed, evidence shows that substantial amounts of income flow into households with solar installations (Winter and Schlesewsky, 2019).

This article analyses whether individuals develop distinct political attitudes as a result of living in households with solar panels over time. Political scientists have long been interested in policy feedback – how the policies of today might affect the politics of tomorrow through changes in, among others, political attitudes. Béland (2010), Campbell (2012) and Larsen (2019) provide systematic reviews of the resulting literature, going as far back as to Schattschneider (1935), with the central argument that ‘new policies create new politics’ (Schattschneider, 1935: 288). Accordingly, public policies, once implemented, shape the politics that create them in the first place.

Pierson (1993) suggests that there might be two mechanisms behind policy feedback on political attitudes among the public. First, policies might alter the allocation of resources in a society, and therefore affect the ability and/or motivation of political actors to get involved in the following political processes. For example, if solar installations generate income, individuals living in solar households will have increased resources – not only money but also time – to be informed about politics. They will also have the motivation to do so because their new income depends on a government programme, and they have a stake in how that programme would look in the future. Similar effects have been recorded elsewhere, such as the Social Security programme in the United States. Campbell (2003) shows that with additional income and early retirement, the recipients of the programme become politically active, which is especially true for the recipients from low-income groups.

Second, public policies might have interpretive effects, changing the way people perceive the world around themselves. In this sense, households with solar installations are likely to learn more about not only the specific government programme but also related issues such as energy transition and climate change. As a result, they might develop political attitudes. For example, Soss (1999) shows that the participants of badly administered programmes can develop mistrust towards governments, taking cues from their interactions with civil servants.

Nevertheless, we know very little about the potential feedback from solar installations. In a recent study, Mildenberger et al. (2019) find that political participation is higher among the members of households with, compared to without, solar installations in the US. The differences between the two types of households are substantively large, with six to nine percentage points higher proportion of solar households voting in elections (Mildenberger, Howe, and Miljanich, 2019: 3). What remains unknown is whether solar installations are the cause of such politically consequential differences. Comparison of households with and without solar panels might be misleading to answer this question, as these groups are likely different from one another in many aspects, some of which might account for the differences in political attitudes – a common problem in the policy feedback literature, with high concerns for reverse causality and omitted variable bias (Campbell, 2012; Larsen, 2019).

Using three panel datasets with repeated measures on households, this article offers a more plausible comparison: changes in political attitudes within individuals over a period of years, between those who start or stop living in a household with solar panels and those whose status do not change during that period. The results show no signs of meaningful effect of solar installations on political attitudes. Individuals living in solar households do not become more or less interested in politics. Neither do they experience a change in their political position, trust in government or party identification.

## Data and Estimation Strategy

To test whether installation of solar panels causes meaningful changes in households’ various political attitudes, I looked for any publicly available, longitudinal study with measures on (a) solar panel ownership, (b) political attitudes and (c) demographic indicators. Three studies met these criteria well: the German Socio-Economic Panel (SOEP), the UK Household Longitudinal Study (UKHLS) and the Swiss Household Panel (SHP). Although the resulting case selection depends on data availability, the credibility of the claims in this analysis is somewhat higher due to the fact that they are based on data from not one but three countries.

Each of these panels is a widely used source of data on households, whose members are surveyed repeatedly over time.^1^ All three datasets include measures of political attitudes as well as demographic indicators, which I use as dependent and control variables, respectively. What makes this selection particularly useful for this study, however, is that these datasets also include indicators for solar panel ownership, the key variable of interest in this study.

The time period varies from one case to another, depending on the survey waves that include the necessary indicators for solar panel ownership. In the SOEP, this occurs in each of the 11 waves between 2007 and 2018, as respondents in these waves are asked whether their dwelling has ‘solar collector, solar energy system’ or not. In the UKHLS, this variable is available for three waves (2008–2009, 2009–2011 and 2012–2014),^2^ in which respondents were asked whether they have installed solar panels for electricity or water heating. For these two datasets, I code *Solar Panel*_it_ as equals to 1 if an individual *i* lives in a dwelling with any kind of solar system at time *t*.

The coding of this variable is less straightforward for the SHP. Beginning with 2013, the household part of the survey indicates whether dwellings have been renovated with solar panels during the previous year. Assuming that once a dwelling is renovated with solar panels, these panels stay in place, I code *Solar Panel*_it_ as 0 if a dwelling has not been renovated with solar panels, as 1 for the renovation year and every year after that until 2018, the latest available wave. To support this assumption, I limit the analysis to those households who have not moved since 2013.

The resulting dataset has 322,309 person-year observations in Germany (68,573 respondents from 39,149 households), 112,570 in the UK (72,869 respondents from 63,988 households) and 79,131 in Switzerland (19,234 respondents from 7885 households).

In terms of outcomes, I focus on a variable that is common across all three datasets for the main part of the analysis: *Political Interest*. This variable measures how interested respondents are in politics, originally with a 5-point scale in the SOEP and UKHLS but with an 11-point scale in the SHP. I have rescaled the variable in the SHP to facilitate comparisons across the cases. As I will also show, the results from this dependent variable do not change if the analysis is on various other variables, including left-right position, trust in government or party identification.

Simple comparisons of political attitudes between those who live in a dwelling with and without solar systems are likely to be misleading. First, there might be systematic differences between these two groups of people, and these differences might affect both solar system adoption and political attitudes. For example, research shows that solar systems are more likely to be adopted by people with high income and high environmental concerns (Jacksohn et al., 2019). At the same time, such characteristics can lead to certain political attitudes. Second, some other important factors, while constant for all individuals, would vary over time. These include, for example, the amount of subsidies of feed-in-tariffs available for solar systems. Similarly, we might observe variations in political attitudes over years, such as increases in political interest in election years.

One way to address these concerns is to use linear fixed-effects regressions in the form of

(1) $$Y_{it}=\beta_{1}\times Solar\;Panel_{it}+\lambda_{i}+\delta_{t}+\epsilon_{it}$$

where $\beta_{1}$ is the causal effect of interest – the effect of solar system ownership on political attitudes, based on within-individual variation associated with starting and/or stopping to live in a dwelling with a solar system during the periods under analysis. In this setting, $\lambda_{i}$ accounts for individual characteristics that do not change over time, while $\delta_{t}$ accounts for changes that evolve from one year to another but are constant across individuals.

I also estimate models with covariates in the form of

(2) $$Y_{it}=\beta_{1}\times Solar\;Panel_{it}+\lambda_{i}+\delta_{t}+\theta \times X_{it}+\epsilon_{it}$$

where $X_{it}$ is a vector of controls for education, head of households, unemployment, household income, households with children, home owners and geographical regions.

## Results

Table 1 presents the regression models of political interest, three models for each country. The first models for each country (Models 1, 4 and 7) are calculated with pooled data – by pooling all individuals across time, without any fixed-effects. For Germany and the UK, the coefficients for *Solar Panel* describe the differences in political interest between those who live in households with solar panels and those who do not. For Switzerland, the comparison is with those who had not acquired solar panels after 2013, who may or may not have renovated their house with solar panels before that year. These coefficients are positive, relatively large, and in the case of Germany and the UK, they are statistically significant. Nevertheless, these results suggest that overall people with solar panels are interested in politics more than those in the comparison groups. In the UK, for example, the differences are about a sixth of a point, over a 4-point scale.

**Table 1.** Effect of Solar System Ownership on Political Interest.

	Germany (1–3)			United Kingdom (4–6)			Switzerland (7–9)		
	(1)	(2)	(3)	(4)	(5)	(6)	(7)	(8)	(9)
Solar panel	0.12^***^ (0.01)	0.01 (0.01)	0.01 (0.01)	0.17^***^ (0.03)	−0.02 (0.03)	−0.02 (0.03)	0.09 (0.08)	−0.004 (0.04)	−0.01 (0.04)
FEs – Individuals	No	Yes	Yes	No	Yes	Yes	No	Yes	Yes
FEs – Waves	No	Yes	Yes	No	Yes	Yes	No	Yes	Yes
Controls	No	No	Yes	No	No	Yes	No	No	Yes
Cl. SEs – Individuals	Yes	No	No	Yes	No	No	Yes	No	No
Cl. SEs – Households	Yes	Yes	Yes	Yes	Yes	Yes	Yes	Yes	Yes
Observations	281,032	281,032	261,253	103,772	103,772	102,831	46,822	46,822	43,352
*R* ^2^	0.002	0.76	0.76	0.0005	0.88	0.88	0.0002	0.86	0.86
Adjusted *R*^2^	0.002	0.69	0.69	0.0005	0.65	0.65	0.0001	0.81	0.81

*Notes*: Standard errors are in parentheses. Control variables include education, head of households, unemployment, household income, households with children, home owners and geographical regions. For variable descriptions, see the Supplemental Appendix.

*** *p* < 0.001.

FEs: fixed effects; SEs: standard errors.

These results, however, are likely to be biased. Those who decide to have solar panels are probably different than those who do not, in various observable and unobservable ways. Moreover, the results from pooled models do not reveal the direction of causality: is political interest causing people to acquire solar panels, or is living in a house with solar panels making people more interested in politics? The fixed-effects models, as specified in Equation 1, are an attempt to address these problems, as these models limit the analysis to within-individuals, comparing changes in individuals that experience a change with regard to solar panels (starting and/or stopping to live in a house with solar panels) to those individuals for whom the solar panel ownership remains constant (i.e. they have always or never lived in solar households during the periods under analysis).

Indeed, when fixed-effects are introduced (Models 2, 5, and 8), we observe a considerable change in the results, compared to the pooled estimates. First, the point estimates become much smaller. In comparison to the pooled models, where the coefficients range between 0.09 and 0.17, with fixed-effects these are reduced to a range from −0.02 to 0.01. These results suggest that after starting to live in a household with solar panels, people might become slightly less as well as slightly more interested in politics. These changes are not only substantively small but also statistically insignificant.

In addition to fixed-effects, the final models of each country include seven control variables as well, controlling for various individual- and household-level factors that change over time, such as household income, as specified in Equation 2. The overall results remain the same: If solar panels affect how much residents in a household are interested in politics, this change is a substantively and statistically insignificant one.

## Sensitivity Analyses

How sensitive are these results to the selection of dependent and control variables? *Political Interest* is only one of the several alternative measures of political attitudes. Would the results change if we analyse a different measure of political attitude? Similarly, the preferred models include all seven control variables together to reduce selection bias, but the results might change if one or more of these control variables are excluded. Therefore, as a sensitivity analysis, I first gather five additional dependent variables from the three datasets, and regress all dependent variables on the mathematical power set of seven control variables. Note that not all dependent variables are available in all three datasets. This exercise results in 1920 regression models.

**Figure 1.**
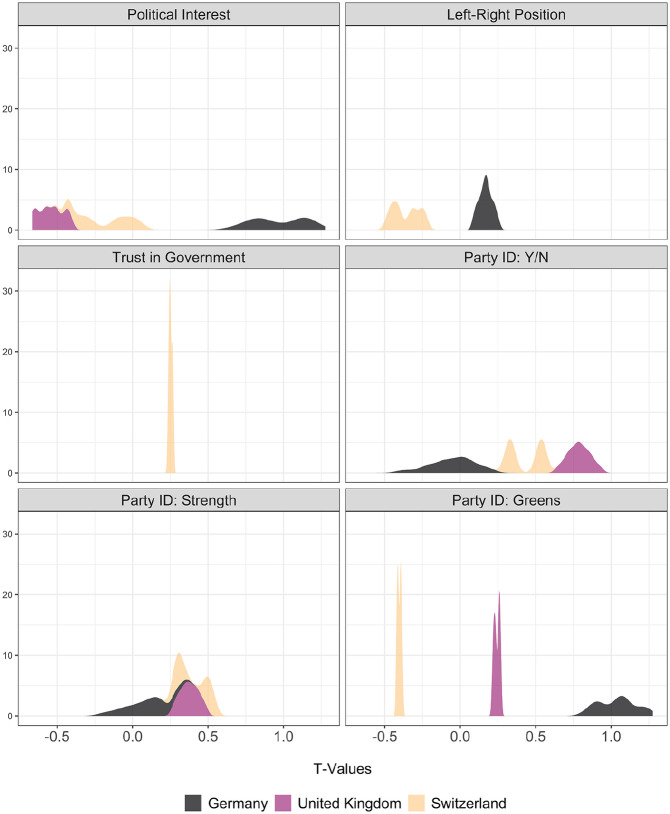
Distribution of *t* Values from Sensitivity Analyses.

Figure 1 plots the *t* values from all possible regression models. In the upper-left box, we see that the results presented in Table 1 are robust to inclusion or exclusion of any of the control variables in the final models: all *t* values are smaller than 2 in absolute value, indicating that the change in *Political Interest* among people who start living under a solar panel is not significantly different from zero than people who experience no such change in their life.

The null results persist for other dependent variables as well. For example, in comparison to respondents who do not acquire solar systems during the time period under analysis, those who do become slightly more or less right-wing, but these changes are not statistically significant. The same is also true if we look at how much these respondents trust in their government or whether and how much they identify with a political party.

A final dependent variable measures political attitudes towards green parties. If solar panels cause changes in environmental attitudes, we are more likely to observe changes in political attitudes towards green parties – parties that own environmental issues. Moreover, solar panels are likely to be more economically beneficial for the owners under a green-party government. However, I find that solar panel ownership does not affect political attitudes towards green parties either: respondents with solar panels are not significantly more likely to identify with green parties. This could be because the solar panel ownership does not seem to change environmental attitudes in the first place, as I show in the Supplemental Appendix.

## Conclusion

Do solar installations have feedback on political attitudes? Individuals living in households with solar installations participate in government programmes with potentially high resource and interpretative effects. The policy feedback theory would therefore predict these individuals to experience changes in terms of attitudes.

To test whether solar installations have feedback over time, I analysed data from three household panels from Germany, Switzerland, and the United Kingdom. This allows testing for any significant changes in political attitudes within individuals over a period of years. Specifically, I compare the attitudes of those who start or stop living in a household with solar panels with those whose ownership status do not change during that period.

The results do not support the expectation that solar installations might have effects on political attitudes. I find that solar installations do not make residents more or less interested in politics – a result that holds if we control for various factors or analyse a set of different outcomes. The latter include respondents’ left-right positions, trust in government and party identification. This is good as well as bad news for actors looking to increase the amount of renewable energy produced through solar installations.

On one hand, it is good news as solar installations with no feedback are less likely to attract opposition from political elites on the losing side of the debate. If individuals living in solar households started identifying with a certain party – say, the green parties – this would create incentives for other parties to oppose the renewable energy programmes for domestic solar energy production.

On the other hand, some might consider the null results as a missed opportunity. One potential outcome of policy feedback is the lock-in effect (Pierson, 1993), where beneficiaries of government programmes act as a political force to consolidate these programmes. If solar installations increased households’ political interest and made them act together for their interest, this could have created incentives for political leaders to invest further resources in solar energy. However, the results in this article suggest that solar installations do not have such effects.

## Supplemental Material

sj-pdf-1-psw-10.1177_14789299211044868 – Supplemental material for Solar Panels and Political Attitudes Click here for additional data file. Supplemental material, sj-pdf-1-psw-10.1177_14789299211044868 for Solar Panels and Political Attitudes by Resul Umit in Political Studies Review

## Notes

1. The data sources are described in detail elsewhere. See, for example, Goebel et al. (2019) for German Socio-Economic Panel (SOEP), Buck and McFall (2011) for UK Household Longitudinal Study (UKHLS) and Tillmann et al. (2016) for Swiss Household Panel (SHP).

2. The first of these waves, 2008–2009, was in fact a part of the British Household Panel Study, which has later been incorporated in to the UKHLS. For more details, see Buck and McFall (2011).
