# Supplementary material for: Solar Panels and Political Attitudes
Source: Polit Stud Rev. 2021 Sep 16;20(3):525–33. doi: 10.1177/14789299211044868 (PMC9340134; doi:10.1177/14789299211044868)
Supplement: sj-pdf-1-psw-10.1177_14789299211044868 – Supplemental material for Solar Panels and Political Attitudes [file sj-pdf-1-psw-10.1177_14789299211044868.pdf]

Online Appendix to

'Solar Panels and Political Attitudes'

Descriptive Statistics

Table A1 presents the summary statistics, by survey wave, for all variables in the main text—except for *Region*, which is a categorical variable. The column *Original* lists the item names in the original datasets that I use to create the variables, for which I provide brief descriptions below. For further details, please see the documentation of the original datasets.

Dependent Variables

Political Interest

- SOEP:
  - Item: 'Generally speaking, how interested are you in politics?'
  - Note: The answer categories—'Very interested,' 'Moderately interested,' 'Not interested,' and 'Disinterested'—are reverse coded so that higher values indicate increasing interest (i.e., 'Disinterested' = 1 . . . 'Very interested' = 4).
- UKHLS:
  - Item: 'How interested would you say you are in politics?'
  - Note: The answer categories—'Very Interested,' 'Fairly interested,' 'Not very interested,' and 'Not at all interested'—are reverse coded so that higher values indicate increasing interest (i.e., 'Not at all interested' = 1 . . . 'Very interested' =

4).

- SHP:
  - Item: ‘Generally, how interested are you in politics, if 0 means “not at all interested” and 10 “very interested?” ’
  - Note: This variable has been rescaled to range from 1 to 4.

Left-Right Position

- SOEP:
  - Item: ‘In politics, people often talk about “left” and “right” when describing different political views. When you think about your own political views, how would you rate them on the scale below? 0: far left, 10: far right.’
- SHP:
  - Item: ‘When they talk about politics, people mention left and right. Personally, where do you position yourself, 0 means “left” and 10 “right?” ’

Trust in Government

- SHP:
  - Item: ‘How much confidence do you have in the federal government (in Bern), if 0 means “no confidence” and 10 means “full confidence?” ’

Party ID: Y/N

- SOEP:
  - Item: ‘Do you lean towards a particular party?’
  - Note: This variable is coded as 0 for ‘No,’ as 1 for ‘Yes.’
- UKHLS:
  - Item: ‘Generally speaking do you think of yourself as a supporter of any one political party?’
  - Note: This variable is coded as 0 for ‘No,’ as 1 for ‘Yes.’

- SHP:
  - Item: 'Overall, do you feel close to any political party?'
  - Note: This variable is coded as 0 for 'No,' as 1 for 'Yes.'

Party ID: Strength

- SOEP:
  - Item: 'To what extent?'
  - Notes. First, the answer categories are reverse coded so that the higher values indicate increasing strength (i.e., 'Very weakly' = 2 ... 'Very strongly' = 6). Then, I have added the code 1 for respondents that do not support any party (i.e., *Party ID* = 0).
- UKHLS:
  - Item: 'Would you call yourself a very strong supporter of [the party that you support], fairly strong or not very strong?'
  - Notes: First, the answer categories are reverse coded so that the values indicate increasing strength (i.e., 'Not very strong' = 2 ... 'Very strong' = 4). Then, I have added the code 1 for respondents that do not support any party (i.e., *Party ID* = 0).
- SHP:
  - Item: 'Do you feel "very close" to this party, "quite close" or "not very close?"'
  - Notes: First, the answer categories are reverse coded so that the values indicate increasing strength (i.e., 'Not very close' = 2 ... 'Very close' = 4). Then, I have added the code 1 for respondents that do not support any party (i.e., *Party ID* = 0).

Party ID: Greens

- SOEP:
  - Item: 'Which party do you lean toward?'

    - Notes: This question is asked only if respondents support a party (i.e., *Party ID* = 1). I code this variable as 1 if they support Alliance90/The Greens, and as 0 if (a) they do not support any party (i.e., *Party ID* = 0) or (b) they support a party other than Alliance90/The Greens. A small number of respondents indicate more than one party. In such cases, I code this variable as 1 if one of these parties are Alliance90/The Greens.

- UKHLS:
    - Item: 'Which political party do you support?'
    - Notes: This question is asked only if respondents support a party (i.e., *Party ID* = 1). I code this variable as 1 if they support the Green Party, and as 0 if (a) they do not support any party (i.e., *Party ID* = 0) or (b) they support a party other than the Green Party.
- SHP:
    - Item: 'Which party do you have in mind?'
    - Notes: This question is asked only if respondents support a party (i.e., *Party ID* = 1). I code this variable as 1 if they support the Green Party of Switzerland, Socialist Green Alternative, or the Green Liberal Party of Switzerland, and as 0 if (a) they do not support any party (i.e., *Party ID* = 0) or (b) they support a party other than the three green parties in Switzerland.

Independent Variable

Solar Panel

- SOEP:
    - Item: 'What amenities does your dwelling have?'
    - Note: This variable is coded as 1 if respondents indicated that their household have a 'Solar collector, solar energy system.' Otherwise it is coded as 0.
- UKHLS:

  - Items: ‘Have you installed or are you seriously considering any of the following…’; ‘solar panels for electricity?’ ‘Solar water heating?’
  - Note: This variable is coded as 1 if respondents answered ‘Yes — fitted’ to either of the questions above. Otherwise it is coded as 0.

- SHP:
  - Item: ‘Have you or your landlord done any larger modernizations in the flat since [month-year]? … What kind of renovations have been made?’
  - Notes: ‘month-year’ refers either to the date the households were last surveyed in the previous year (for existing households) or to a date 12 months earlier (for new households). I have coded this variable as 0 if respondents replied ‘No’ to solar-panel modernisations. If they replied ‘Yes,’ I have coded this variable as 1 for that survey wave *and* for any other wave that follows.

Control Variables

Education

- SOEP:
  - Item: ‘ISCED-1997-Classification’
  - Note: This variable ranges between ‘In school’ = 0 to ‘Higher education’ = 6.
- UKHLS:
  - Item: ‘Current status: highest educational or vocational qualification’
  - Note: This variable is reverse coded so that it ranges between ‘No qualification’ = 1 and ‘University degree’ = 6.
- SHP:
  - Item: ‘International standard classification of education ISCED 1997’
  - Note: This variable has been recoded to range between ‘Not completed primary (compulsory) education’ = 0 to ‘Second stage of tertiary education’ = 9.

Head of Household

- SOEP:
  - Item: 'Relationship to the head of household'
  - Note: This variable is coded as 1 for the answer category 'Head Of HH, Contact Person,' as 0 for other household members.
- UKHLS:
  - Items: 'Conventional head of household indicator' (Wave 2008-2009); 'Household reference person' (Waves 2009-2011 and 2012-2014).
  - This variable is coded as 1 for respondents who are 'Head of household' (Wave 2008-2009) or 'Reference person' (Waves 2009-2011 and 2012-2014). Otherwise it is coded as 0.
- SHP:
  - Item: 'Relation to the person of reference'
  - Note: This variable is coded as 1 for 'Reference person,' as 0 for other household members.

Unemployed

- SOEP:
  - Item: 'Occupational status'
  - Note: This variable is coded as 1 if respondents are 'Unemployed, Not Employer.' Otherwise it is coded as 0.
- UKHLS:
  - Item: 'Which of these best describes your current employment situation?'
  - Note: This variable is coded as 1 if respondents are 'Unemployed.' Otherwise it is coded as 0.
- SHP:
  - Item: 'Working status'

- Note: This variable is coded as 1 if respondents are ‘Unemployed.’ Otherwise it is coded as 0.

Household Income

- SOEP:
  - Item: ‘Monthly household net income’
- UKHLS:
  - Items: ‘Annual household net income’ (Wave 2008-2009); ‘Monthly household net income’ (Waves 2009-2011 and 2012-2014)
  - Note: For the Wave 2008-2009, I have divided the annual household net income into 12 so that all values refer to monthly income.
- SHP:
  - Item: ‘Yearly household income net’
  - Note: I have divided the original values into 12 so that all values refer to monthly income.

Household with Children

- SOEP:
  - Item: ‘Children under 16 years in household’
  - Note: This variable is coded as 1 if there are one or more children under 16 years in household. Otherwise it is coded as 0.
- UKHLS:
  - Item: ‘Number of children in household’
  - Note: This item counts the children aged 15 or under. I have coded this variable as 0 for households with no children. Otherwise it is coded as 1.
- SHP:
  - Item: ‘Number of children in household: 0 to 17 years’
  - Note: This variable is coded as 1 for households with one or more children.

Otherwise it is coded as 0.

House Owned

- SOEP:
  - Item: 'Tenant or owner of dwelling'
  - Note: This variable is coded as 1 for owners. Otherwise it is coded as 0.
- UKHLS:
  - Item: 'Does your household own this accommodation outright, is it being bought with a mortgage, is it rented or does it come rent-free?'
  - Note: This variable is coded as 1 for properties that are owned, owned with mortgage, or those that involve shared ownership. Otherwise it is coded as 0.
- SHP:
  - Item: 'Are you, or another person living in your household, a tenant or owner of the accommodation you currently live in?'
  - Note: This variable is coded as 1 for those indicating 'Owner/co-owner.' Otherwise it is coded as 0.

Region

- SOEP:
  - Item: 'NUTS-systematic-1 (federal state)'
  - Note: This is a categorical variable, with 16 levels. It refers to the *NUTS 1* regions of Germany.
- UKHLS:
  - Item: 'Government office region'
  - Notes: This is a categorical variable, with 12 levels. It refers to the *NUTS 1* regions of the UK.
- SHP:
  - Item: 'Region of residence'

– Note: This is a categorical variable, with 7 levels. It refers to the *NUTS 2* regions of Switzerland.

Table A1: Summary Statistics by Survey Wave

Dataset	Variable	Wave	Origin	N	Mean	SD	Median	Min	Max
SOEP	Solar Panel	2007	hlf0035	20216	0.05	0.23	0.000	0.00	1.00
SOEP	Solar Panel	2008	hlf0035	19034	0.06	0.24	0.000	0.00	1.00
SOEP	Solar Panel	2009	hlf0035	20390	0.08	0.26	0.000	0.00	1.00
SOEP	Solar Panel	2010	hlf0035	26244	0.09	0.28	0.000	0.00	1.00
SOEP	Solar Panel	2011	hlf0035	28285	0.10	0.30	0.000	0.00	1.00
SOEP	Solar Panel	2012	hlf0035	27661	0.11	0.31	0.000	0.00	1.00
SOEP	Solar Panel	2013	hlf0035	30772	0.10	0.30	0.000	0.00	1.00
SOEP	Solar Panel	2014	hlf0035	27178	0.11	0.31	0.000	0.00	1.00
SOEP	Solar Panel	2015	hlf0035	26840	0.11	0.32	0.000	0.00	1.00
SOEP	Solar Panel	2016	hlf0035	24393	0.11	0.32	0.000	0.00	1.00
SOEP	Solar Panel	2017	hlf0035	26670	0.12	0.33	0.000	0.00	1.00
SOEP	Solar Panel	2018	hlf0035	25562	0.12	0.33	0.000	0.00	1.00
SOEP	Political Interest	2007	plh0007	20841	2.32	0.80	2.000	1.00	4.00
SOEP	Political Interest	2008	plh0007	19624	2.27	0.81	2.000	1.00	4.00
SOEP	Political Interest	2009	plh0007	20728	2.29	0.85	2.000	1.00	4.00
SOEP	Political Interest	2010	plh0007	26632	2.33	0.81	2.000	1.00	4.00
SOEP	Political Interest	2011	plh0007	21030	2.36	0.84	2.000	1.00	4.00
SOEP	Political Interest	2012	plh0007	20773	2.35	0.81	2.000	1.00	4.00
SOEP	Political Interest	2013	plh0007	24075	2.27	0.84	2.000	1.00	4.00

Table A1: Summary Statistics by Survey Wave (*continued*)

Dataset	Variable	Wave	Origin	N	Mean	SD	Median	Min	Max
SOEP	Political Interest	2014	plh0007	27419	2.34	0.82	2.000	1.00	4.00
SOEP	Political Interest	2015	plh0007	27109	2.26	0.84	2.000	1.00	4.00
SOEP	Political Interest	2016	plh0007	28977	2.23	0.87	2.000	1.00	4.00
SOEP	Political Interest	2017	plh0007	32385	2.22	0.89	2.000	1.00	4.00
SOEP	Political Interest	2018	plh0007	30236	2.25	0.88	2.000	1.00	4.00
SOEP	Left-Right Position	2009	plh0004	19965	4.76	1.65	5.000	0.00	10.00
SOEP	Left-Right Position	2014	plh0004	25903	4.70	1.59	5.000	0.00	10.00
SOEP	Party ID: Y/N	2007	plh0011_h	20798	0.43	0.50	0.000	0.00	1.00
SOEP	Party ID: Y/N	2008	plh0011_h	19613	0.44	0.50	0.000	0.00	1.00
SOEP	Party ID: Y/N	2009	plh0011_h	20680	0.44	0.50	0.000	0.00	1.00
SOEP	Party ID: Y/N	2010	plh0011_h	26583	0.47	0.50	0.000	0.00	1.00
SOEP	Party ID: Y/N	2011	plh0011_h	20942	0.45	0.50	0.000	0.00	1.00
SOEP	Party ID: Y/N	2012	plh0011_h	20709	0.44	0.50	0.000	0.00	1.00
SOEP	Party ID: Y/N	2013	plh0011_h	23928	0.42	0.49	0.000	0.00	1.00
SOEP	Party ID: Y/N	2014	plh0011_h	27226	0.46	0.50	0.000	0.00	1.00
SOEP	Party ID: Y/N	2015	plh0011_h	26935	0.40	0.49	0.000	0.00	1.00
SOEP	Party ID: Y/N	2016	plh0011_h	24521	0.40	0.49	0.000	0.00	1.00
SOEP	Party ID: Y/N	2017	plh0011_h	29342	0.39	0.49	0.000	0.00	1.00
SOEP	Party ID: Y/N	2018	plh0011_h	29602	0.40	0.49	0.000	0.00	1.00

Table A1: Summary Statistics by Survey Wave (*continued*)

Dataset	Variable	Wave	Origin	N	Mean	SD	Median	Min	Max
SOEP	Party ID: Strength	2007	plh0013_h	20342	2.43	1.74	1.000	1.00	6.00
SOEP	Party ID: Strength	2008	plh0013_h	19220	2.45	1.76	1.000	1.00	6.00
SOEP	Party ID: Strength	2009	plh0013_h	20162	2.46	1.77	1.000	1.00	6.00
SOEP	Party ID: Strength	2010	plh0013_h	25920	2.57	1.78	1.000	1.00	6.00
SOEP	Party ID: Strength	2011	plh0013_h	20416	2.51	1.79	1.000	1.00	6.00
SOEP	Party ID: Strength	2012	plh0013_h	20229	2.47	1.76	1.000	1.00	6.00
SOEP	Party ID: Strength	2013	plh0013_h	23461	2.40	1.75	1.000	1.00	6.00
SOEP	Party ID: Strength	2014	plh0013_h	26526	2.52	1.77	1.000	1.00	6.00
SOEP	Party ID: Strength	2015	plh0013_h	26377	2.33	1.73	1.000	1.00	6.00
SOEP	Party ID: Strength	2016	plh0013_h	24160	2.34	1.74	1.000	1.00	6.00
SOEP	Party ID: Strength	2017	plh0013_h	28299	2.29	1.75	1.000	1.00	6.00
SOEP	Party ID: Strength	2018	plh0013_h	28301	2.27	1.73	1.000	1.00	6.00
SOEP	Party ID: Greens	2007	plh0012_h	8934	0.10	0.31	0.000	0.00	1.00
SOEP	Party ID: Greens	2008	plh0012_h	8470	0.10	0.30	0.000	0.00	1.00
SOEP	Party ID: Greens	2009	plh0012_h	8879	0.11	0.32	0.000	0.00	1.00
SOEP	Party ID: Greens	2010	plh0012_h	12164	0.15	0.36	0.000	0.00	1.00
SOEP	Party ID: Greens	2011	plh0012_h	9197	0.18	0.38	0.000	0.00	1.00
SOEP	Party ID: Greens	2012	plh0012_h	8986	0.16	0.36	0.000	0.00	1.00
SOEP	Party ID: Greens	2013	plh0012_h	9776	0.16	0.36	0.000	0.00	1.00

Table A1: Summary Statistics by Survey Wave (*continued*)

Dataset	Variable	Wave	Origin	N	Mean	SD	Median	Min	Max
SOEP	Party ID: Greens	2014	plh0012_h	12042	0.15	0.36	0.000	0.00	1.00
SOEP	Party ID: Greens	2015	plh0012_h	10400	0.15	0.36	0.000	0.00	1.00
SOEP	Party ID: Greens	2016	plh0012_h	9530	0.16	0.37	0.000	0.00	1.00
SOEP	Party ID: Greens	2017	plh0012_h	11011	0.13	0.34	0.000	0.00	1.00
SOEP	Party ID: Greens	2018	plh0012_h	11227	0.16	0.37	0.000	0.00	1.00
SOEP	Education	2007	pgisced97	20540	3.68	1.47	3.000	0.00	6.00
SOEP	Education	2008	pgisced97	19338	3.69	1.48	3.000	0.00	6.00
SOEP	Education	2009	pgisced97	20439	3.70	1.48	3.000	0.00	6.00
SOEP	Education	2010	pgisced97	26372	3.70	1.48	3.000	0.00	6.00
SOEP	Education	2011	pgisced97	28262	3.69	1.48	3.000	0.00	6.00
SOEP	Education	2012	pgisced97	27548	3.70	1.48	3.000	0.00	6.00
SOEP	Education	2013	pgisced97	30388	3.65	1.53	3.000	0.00	6.00
SOEP	Education	2014	pgisced97	26827	3.70	1.51	3.000	0.00	6.00
SOEP	Education	2015	pgisced97	26510	3.74	1.52	3.000	0.00	6.00
SOEP	Education	2016	pgisced97	28161	3.59	1.62	3.000	0.00	6.00
SOEP	Education	2017	pgisced97	31523	3.56	1.64	3.000	0.00	6.00
SOEP	Education	2018	pgisced97	29150	3.60	1.62	3.000	0.00	6.00
SOEP	Head of Household	2007	stell_h	20886	0.56	0.50	1.000	0.00	1.00
SOEP	Head of Household	2008	stell_h	19684	0.56	0.50	1.000	0.00	1.00

Table A1: Summary Statistics by Survey Wave (*continued*)

Dataset	Variable	Wave	Origin	N	Mean	SD	Median	Min	Max
SOEP	Head of Household	2009	stell_h	20792	0.57	0.50	1.000	0.00	1.00
SOEP	Head of Household	2010	stell_h	26720	0.58	0.49	1.000	0.00	1.00
SOEP	Head of Household	2011	stell_h	28733	0.59	0.49	1.000	0.00	1.00
SOEP	Head of Household	2012	stell_h	27983	0.59	0.49	1.000	0.00	1.00
SOEP	Head of Household	2013	stell_h	30956	0.58	0.49	1.000	0.00	1.00
SOEP	Head of Household	2014	stell_h	27465	0.58	0.49	1.000	0.00	1.00
SOEP	Head of Household	2015	stell_h	27183	0.59	0.49	1.000	0.00	1.00
SOEP	Head of Household	2016	stell_h	29116	0.61	0.49	1.000	0.00	1.00
SOEP	Head of Household	2017	stell_h	32485	0.60	0.49	1.000	0.00	1.00
SOEP	Head of Household	2018	stell_h	30306	0.61	0.49	1.000	0.00	1.00
SOEP	Unemployed	2007	pgstib	20861	0.06	0.23	0.000	0.00	1.00
SOEP	Unemployed	2008	pgstib	19648	0.05	0.22	0.000	0.00	1.00
SOEP	Unemployed	2009	pgstib	20729	0.05	0.22	0.000	0.00	1.00
SOEP	Unemployed	2010	pgstib	26625	0.07	0.25	0.000	0.00	1.00
SOEP	Unemployed	2011	pgstib	28605	0.06	0.24	0.000	0.00	1.00
SOEP	Unemployed	2012	pgstib	27925	0.06	0.23	0.000	0.00	1.00
SOEP	Unemployed	2013	pgstib	30864	0.07	0.25	0.000	0.00	1.00
SOEP	Unemployed	2014	pgstib	27395	0.06	0.24	0.000	0.00	1.00
SOEP	Unemployed	2015	pgstib	27085	0.06	0.25	0.000	0.00	1.00

Table A1: Summary Statistics by Survey Wave (*continued*)

Dataset	Variable	Wave	Origin	N	Mean	SD	Median	Min	Max
SOEP	Unemployed	2016	pgstib	28977	0.05	0.22	0.000	0.00	1.00
SOEP	Unemployed	2017	pgstib	32318	0.08	0.27	0.000	0.00	1.00
SOEP	Unemployed	2018	pgstib	30096	0.09	0.29	0.000	0.00	1.00
SOEP	Household Income	2007	hghinc	19824	2777.23	1879.08	2400.000	0.00	42667.00
SOEP	Household Income	2008	hghinc	18578	2831.84	2127.72	2500.000	0.00	99999.00
SOEP	Household Income	2009	hghinc	19440	2831.63	1872.40	2500.000	0.00	30000.00
SOEP	Household Income	2010	hghinc	24992	2846.58	1861.41	2500.000	0.00	35000.00
SOEP	Household Income	2011	hghinc	27427	2896.88	1916.77	2500.000	40.00	55000.00
SOEP	Household Income	2012	hghinc	26718	2978.51	2699.70	2500.000	0.00	200000.00
SOEP	Household Income	2013	hghinc	29512	2963.65	1982.67	2530.000	0.00	70000.00
SOEP	Household Income	2014	hghinc	26263	3067.32	2030.76	2700.000	150.00	65000.00
SOEP	Household Income	2015	hghinc	25813	3108.73	1958.88	2700.000	0.00	60000.00
SOEP	Household Income	2016	hghinc	27679	2898.75	2081.08	2500.000	0.00	45000.00
SOEP	Household Income	2017	hghinc	30867	3052.78	8308.01	2600.000	88.00	999999.00
SOEP	Household Income	2018	hghinc	28549	3131.94	2147.27	2800.000	100.00	60000.00
SOEP	Children in Household	2007	hlk0044	20869	0.27	0.44	0.000	0.00	1.00
SOEP	Children in Household	2008	hlk0044	19669	0.26	0.44	0.000	0.00	1.00
SOEP	Children in Household	2009	hlk0044	20777	0.25	0.43	0.000	0.00	1.00
SOEP	Children in Household	2010	hlk0044	26694	0.46	0.50	0.000	0.00	1.00

Table A1: Summary Statistics by Survey Wave (*continued*)

Dataset	Variable	Wave	Origin	N	Mean	SD	Median	Min	Max
SOEP	Children in Household	2011	hlk0044	28691	0.43	0.50	0.000	0.00	1.00
SOEP	Children in Household	2012	hlk0044	27940	0.41	0.49	0.000	0.00	1.00
SOEP	Children in Household	2013	hlk0044	30934	0.43	0.49	0.000	0.00	1.00
SOEP	Children in Household	2014	hlk0044	27367	0.40	0.49	0.000	0.00	1.00
SOEP	Children in Household	2015	hlk0044	27108	0.40	0.49	0.000	0.00	1.00
SOEP	Children in Household	2016	hlk0044	28993	0.42	0.49	0.000	0.00	1.00
SOEP	Children in Household	2017	hlk0044	32397	0.40	0.49	0.000	0.00	1.00
SOEP	Children in Household	2018	hlk0044	25869	0.35	0.48	0.000	0.00	1.00
SOEP	House Owned	2007	hgowner	20869	0.54	0.50	1.000	0.00	1.00
SOEP	House Owned	2008	hgowner	19669	0.55	0.50	1.000	0.00	1.00
SOEP	House Owned	2009	hgowner	20777	0.55	0.50	1.000	0.00	1.00
SOEP	House Owned	2010	hgowner	26694	0.51	0.50	1.000	0.00	1.00
SOEP	House Owned	2011	hgowner	28691	0.52	0.50	1.000	0.00	1.00
SOEP	House Owned	2012	hgowner	27940	0.52	0.50	1.000	0.00	1.00
SOEP	House Owned	2013	hgowner	30934	0.47	0.50	0.000	0.00	1.00
SOEP	House Owned	2014	hgowner	27370	0.49	0.50	0.000	0.00	1.00
SOEP	House Owned	2015	hgowner	27108	0.47	0.50	0.000	0.00	1.00
SOEP	House Owned	2016	hgowner	24558	0.48	0.50	0.000	0.00	1.00
SOEP	House Owned	2017	hgowner	30145	0.44	0.50	0.000	0.00	1.00

Table A1: Summary Statistics by Survey Wave (*continued*)

Dataset	Variable	Wave	Origin	N	Mean	SD	Median	Min	Max
SOEP	House Owned	2018	hgowner	30220	0.41	0.49	0.000	0.00	1.00
UKLHS	Solar Panel	2008-2009	br_solar1/2	13607	0.01	0.08	0.000	0.00	1.00
UKLHS	Solar Panel	2009-2011	a_solar1/2	50837	0.01	0.10	0.000	0.00	1.00
UKLHS	Solar Panel	2012-2014	d_solar1/2	47039	0.03	0.16	0.000	0.00	1.00
UKLHS	Political Interest	2008-2009	br_vote6	14186	2.20	0.93	2.000	1.00	4.00
UKLHS	Political Interest	2009-2011	a_vote6	47530	2.27	0.97	2.000	1.00	4.00
UKLHS	Political Interest	2012-2014	d_vote6	43114	2.21	0.98	2.000	1.00	4.00
UKLHS	Party ID: Y/N	2008-2009	br_vote1	14186	0.35	0.48	0.000	0.00	1.00
UKLHS	Party ID: Y/N	2009-2011	a_vote1	47493	0.31	0.46	0.000	0.00	1.00
UKLHS	Party ID: Y/N	2012-2014	d_vote1	43084	0.30	0.46	0.000	0.00	1.00
UKLHS	Party Id: Strength	2008-2009	br_vote5	14078	1.57	0.88	1.000	1.00	4.00
UKLHS	Party Id: Strength	2009-2011	a_vote5	47155	1.53	0.89	1.000	1.00	4.00
UKLHS	Party Id: Strength	2012-2014	d_vote5	42902	1.50	0.86	1.000	1.00	4.00
UKLHS	Party ID: Greens	2008-2009	br_vote4	14134	0.01	0.08	0.000	0.00	1.00
UKLHS	Party ID: Greens	2009-2011	a_vote4	47156	0.01	0.10	0.000	0.00	1.00
UKLHS	Party ID: Greens	2012-2014	d_vote4	42910	0.01	0.10	0.000	0.00	1.00
UKLHS	Education	2008-2009	br_hiqualb_dv	13873	3.40	1.62	3.000	1.00	6.00
UKLHS	Education	2009-2011	a_hiqualb_dv	50902	3.59	1.74	4.000	1.00	6.00
UKLHS	Education	2012-2014	d_hiqualb_dv	46937	3.75	1.68	4.000	1.00	6.00

Table A1: Summary Statistics by Survey Wave (*continued*)

Dataset	Variable	Wave	Origin	N	Mean	SD	Median	Min	Max
UKLHS	Head of Household	2008-2009	br_hoh	14419	0.52	0.50	1.000	0.00	1.00
UKLHS	Head of Household	2009-2011	a_hrpid	50994	0.54	0.50	1.000	0.00	1.00
UKLHS	Head of Household	2012-2014	d_hrpid	47157	0.52	0.50	1.000	0.00	1.00
UKLHS	Unemployed	2008-2009	br_jbstat	14418	0.03	0.18	0.000	0.00	1.00
UKLHS	Unemployed	2009-2011	a_jbstat	50982	0.07	0.25	0.000	0.00	1.00
UKLHS	Unemployed	2012-2014	d_jbstat	47152	0.05	0.22	0.000	0.00	1.00
UKLHS	Household Income	2008-2009	br_hhyneti	13683	2389.43	1517.73	2116.013	0.00	24215.51
UKLHS	Household Income	2009-2011	a_fihhmnnet1_dv	50952	2997.57	30906.76	2235.659	0.00	4001937.50
UKLHS	Household Income	2012-2014	d_fihhmnnet1_dv	47126	3246.65	4709.97	2642.720	0.00	199900.00
UKLHS	Children in Household	2008-2009	br_nkids_dv	14419	0.32	0.47	0.000	0.00	1.00
UKLHS	Children in Household	2009-2011	a_nkids_dv	50994	0.35	0.48	0.000	0.00	1.00
UKLHS	Children in Household	2012-2014	d_nkids_dv	47157	0.34	0.47	0.000	0.00	1.00
UKLHS	House Owned	2008-2009	br_hsownd_bh	14318	0.76	0.43	1.000	0.00	1.00
UKLHS	House Owned	2009-2011	a_hsownd_bh	50863	0.66	0.47	1.000	0.00	1.00
UKLHS	House Owned	2012-2014	d_hsownd_bh	46723	0.72	0.45	1.000	0.00	1.00
SHP	Solar Panel	2013	h13h44	17533	0.00	0.06	0.000	0.00	1.00
SHP	Solar Panel	2014	h14h44	14964	0.01	0.09	0.000	0.00	1.00
SHP	Solar Panel	2015	h15h44	13288	0.01	0.11	0.000	0.00	1.00
SHP	Solar Panel	2016	h16h44	11978	0.01	0.12	0.000	0.00	1.00

Table A1: Summary Statistics by Survey Wave (*continued*)

Dataset	Variable	Wave	Origin	N	Mean	SD	Median	Min	Max
SHP	Solar Panel	2017	h17h44	10878	0.02	0.13	0.000	0.00	1.00
SHP	Solar Panel	2018	h18h44	10490	0.02	0.15	0.000	0.00	1.00
SHP	Political Interest	2013	p13p01	5808	2.64	0.85	2.800	1.00	4.00
SHP	Political Interest	2014	p14p01	9803	2.60	0.85	2.800	1.00	4.00
SHP	Political Interest	2015	p15p01	8904	2.66	0.83	2.800	1.00	4.00
SHP	Political Interest	2016	p16p01	7917	2.67	0.85	2.800	1.00	4.00
SHP	Political Interest	2017	p17p01	7294	2.57	0.87	2.500	1.00	4.00
SHP	Political Interest	2018	p18p01	7096	2.67	0.83	2.800	1.00	4.00
SHP	Left-Right Position	2013	p13p10	5052	5.01	2.12	5.000	0.00	10.00
SHP	Left-Right Position	2014	p14p10	8492	5.04	2.16	5.000	0.00	10.00
SHP	Left-Right Position	2015	p15p10	7792	5.04	2.19	5.000	0.00	10.00
SHP	Left-Right Position	2016	p16p10	6992	4.97	2.19	5.000	0.00	10.00
SHP	Left-Right Position	2017	p17p10	6433	4.92	2.20	5.000	0.00	10.00
SHP	Left-Right Position	2018	p18p10	6254	4.88	2.09	5.000	0.00	10.00
SHP	Trust in Government	2014	p14p04	5626	6.00	2.06	6.000	0.00	10.00
SHP	Trust in Government	2017	p17p04	7193	6.34	1.99	7.000	0.00	10.00
SHP	Party ID: Y/N	2014	p14p66	5646	0.31	0.46	0.000	0.00	1.00
SHP	Party ID: Y/N	2017	p17p66	7230	0.31	0.46	0.000	0.00	1.00
SHP	Party ID: Strength	2014	p14p68	5628	1.56	0.90	1.000	1.00	4.00

Table A1: Summary Statistics by Survey Wave (*continued*)

Dataset	Variable	Wave	Origin	N	Mean	SD	Median	Min	Max
SHP	Party ID: Strength	2017	p17p68	7207	1.57	0.91	1.000	1.00	4.00
SHP	Party ID: Greens	2014	p14p67	1726	0.14	0.35	0.000	0.00	1.00
SHP	Party ID: Greens	2017	p17p67	2250	0.14	0.34	0.000	0.00	1.00
SHP	Education	2013	isced13	8808	4.18	2.46	4.000	0.00	9.00
SHP	Education	2014	isced14	14912	4.18	2.46	4.000	0.00	9.00
SHP	Education	2015	isced15	13244	4.23	2.46	4.000	0.00	9.00
SHP	Education	2016	isced16	11938	4.29	2.45	4.000	0.00	9.00
SHP	Education	2017	isced17	10838	4.34	2.44	4.000	0.00	9.00
SHP	Education	2018	isced18	10445	4.38	2.43	4.000	0.00	9.00
SHP	Head of Household	2013	relarp13	17521	0.42	0.49	0.000	0.00	1.00
SHP	Head of Household	2014	relarp14	14963	0.41	0.49	0.000	0.00	1.00
SHP	Head of Household	2015	relarp15	13286	0.42	0.49	0.000	0.00	1.00
SHP	Head of Household	2016	relarp16	11974	0.42	0.49	0.000	0.00	1.00
SHP	Head of Household	2017	relarp17	10876	0.43	0.50	0.000	0.00	1.00
SHP	Head of Household	2018	relarp18	10490	0.44	0.50	0.000	0.00	1.00
SHP	Unemployed	2013	wstat13	6017	0.02	0.13	0.000	0.00	1.00
SHP	Unemployed	2014	wstat14	10138	0.01	0.12	0.000	0.00	1.00
SHP	Unemployed	2015	wstat15	9196	0.02	0.13	0.000	0.00	1.00
SHP	Unemployed	2016	wstat16	8167	0.02	0.12	0.000	0.00	1.00

Table A1: Summary Statistics by Survey Wave (*continued*)

Dataset	Variable	Wave	Origin	N	Mean	SD	Median	Min	Max
SHP	Unemployed	2017	wstat17	7523	0.02	0.12	0.000	0.00	1.00
SHP	Unemployed	2018	wstat18	7306	0.01	0.12	0.000	0.00	1.00
SHP	Household Income	2013	i13htyn	14884	9650.78	6553.53	8500.000	0.00	183729.17
SHP	Household Income	2014	i14htyn	13377	10412.98	7198.26	9166.670	0.00	218702.50
SHP	Household Income	2015	i15htyn	11949	10349.85	6237.54	9216.670	60.83	153000.00
SHP	Household Income	2016	i16htyn	10733	10427.05	6417.87	9223.330	127.50	125333.33
SHP	Household Income	2017	i17htyn	9771	10420.52	6447.76	9250.000	150.00	93900.00
SHP	Household Income	2018	i18htyn	9417	10520.81	6687.09	9323.330	260.83	103550.83
SHP	Children in Household	2013	nbkid13	17533	0.43	0.49	0.000	0.00	1.00
SHP	Children in Household	2014	nbkid14	14964	0.42	0.49	0.000	0.00	1.00
SHP	Children in Household	2015	nbkid15	13288	0.40	0.49	0.000	0.00	1.00
SHP	Children in Household	2016	nbkid16	11978	0.38	0.49	0.000	0.00	1.00
SHP	Children in Household	2017	nbkid17	10878	0.37	0.48	0.000	0.00	1.00
SHP	Children in Household	2018	nbkid18	10490	0.35	0.48	0.000	0.00	1.00
SHP	House Owned	2013	h13h29	17166	0.61	0.49	1.000	0.00	1.00
SHP	House Owned	2014	h14h29	14620	0.64	0.48	1.000	0.00	1.00
SHP	House Owned	2015	h15h29	13089	0.65	0.48	1.000	0.00	1.00
SHP	House Owned	2016	h16h29	11689	0.66	0.47	1.000	0.00	1.00
SHP	House Owned	2017	h17h29	10644	0.65	0.48	1.000	0.00	1.00

Table A1: Summary Statistics by Survey Wave (*continued*)

Dataset	Variable	Wave	Origin	N	Mean	SD	Median	Min	Max
SHP	House Owned	2018	h18h29	10320	0.65	0.48	1.000	0.00	1.00

Robustness Checks

Subgroups: House Owners and Heads of Households

Table A2: Models of political interest—interaction with *House Owned*

	Germany (1 - 3)			United Kingdom (4 - 6)			Switzerland (7 - 9)		
	(1)	(2)	(3)	(4)	(5)	(6)	(7)	(8)	(9)
Solar Panel (SP)	0.07**	0.004	0.004	−0.002	0.02	0.02	0.01	0.05	0.04
	(0.02)	(0.01)	(0.01)	(0.08)	(0.09)	(0.09)	(0.19)	(0.09)	(0.10)
House Owned (HO)	0.19***	−0.01	−0.01	0.32***	0.02	0.02	0.16***	0.01	0.01
	(0.01)	(0.01)	(0.01)	(0.01)	(0.02)	(0.02)	(0.02)	(0.02)	(0.02)
SP x HO	−0.02	0.01	0.005	0.14	−0.05	−0.05	0.07	−0.06	−0.06
	(0.03)	(0.02)	(0.02)	(0.08)	(0.10)	(0.10)	(0.21)	(0.09)	(0.10)
FEs—Individuals	No	Yes	Yes	No	Yes	Yes	No	Yes	Yes
FEs—Waves	No	Yes	Yes	No	Yes	Yes	No	Yes	Yes
Controls	No	No	Yes	No	No	Yes	No	No	Yes
Cl. SEs—Individuals	Yes	No	No	Yes	No	No	Yes	No	No
Cl. SEs—Households	Yes	Yes	Yes	Yes	Yes	Yes	Yes	Yes	Yes
Observations	281,031	281,031	261,253	103,349	103,349	102,831	46,717	46,717	43,352
R^2	0.01	0.76	0.76	0.02	0.88	0.88	0.01	0.86	0.86
Adjusted R^2	0.01	0.69	0.69	0.02	0.65	0.65	0.01	0.81	0.81

Notes: Standard errors are in parentheses. Control variables include education, head of households, unemployment, household income, households with children, house owners, and geographical regions. * $p < 0.05$, ** $p < 0.01$, *** $p < 0.001$.

Would the null results change if we analyse relevant subgroups, such as those who live in a house that they own or those who are heads of households? These groups of individuals are more likely to benefit, and therefore be affected, from living in a house with solar installations. To explore the possibility that the results might be heterogeneous with regard to these factors, I estimate interactions effects, between *Solar Panel* and *House Owner* (Table A2) and between *Solar Panel* and *Head of Household* (Table A3), for each of the three models in Table 1.

The results show no signs of heterogeneity, with the interaction terms returning substantively and statistically insignificant estimates in all models. This suggests that the null results continue to hold if we limit the analysis to house owners or tenants, heads or other members

of households.

Table A3: Models of political interest—interaction with *Head of Household*

	Germany (1 - 3)			United Kingdom (4 - 6)			Switzerland (7 - 9)		
	(1)	(2)	(3)	(4)	(5)	(6)	(7)	(8)	(9)
Solar Panel (SP)	0.12***	0.02	0.01	0.15***	−0.02	−0.02	0.15	0.03	0.02
	(0.02)	(0.01)	(0.01)	(0.04)	(0.05)	(0.05)	(0.10)	(0.05)	(0.05)
Head of Household (HH)	0.18***	0.01	0.01	0.18***	−0.001	−0.003	0.14***	−0.01	−0.01
	(0.01)	(0.01)	(0.01)	(0.01)	(0.01)	(0.01)	(0.01)	(0.01)	(0.01)
SP x HH	0.04*	−0.01	−0.01	0.05	0.002	0.01	−0.10	−0.05	−0.06
	(0.02)	(0.01)	(0.01)	(0.05)	(0.06)	(0.06)	(0.08)	(0.06)	(0.06)
FEs—Individuals	No	Yes	Yes	No	Yes	Yes	No	Yes	Yes
FEs—Waves	No	Yes	Yes	No	Yes	Yes	No	Yes	Yes
Controls	No	No	Yes	No	No	Yes	No	No	Yes
Cl. SEs—Individuals	Yes	No	No	Yes	No	No	Yes	No	No
Cl. SEs—Households	Yes	Yes	Yes	Yes	Yes	Yes	Yes	Yes	Yes
Observations	281,032	281,032	261,254	103,772	103,772	103,252	46,819	46,819	43,380
R^2	0.01	0.76	0.76	0.01	0.88	0.88	0.01	0.86	0.86
Adjusted R^2	0.01	0.69	0.69	0.01	0.65	0.65	0.01	0.81	0.81

Notes: Standard errors are in parentheses. Control variables include education, head of households, unemployment, household income, households with children, house owners, and geographical regions. * $p < 0.05$, ** $p < 0.01$, *** $p < 0.001$.

Solar System Types

There are two main types of solar systems: solar photovoltaic (PV) and solar thermal (ST). PV systems produce electricity, which can be consumed in the dwelling and/or fed back into the electricity grid for compensation from the utility. In comparison, ST systems turn sunlight into heat, providing warm water or space heating for the dwelling. It is also possible to have both systems on the same rooftop.

One concern is that the effects of solar systems might be heterogeneous, depending on the type of solar systems that households have on their rooftop perhaps because they generate different amounts of revenues. For example, Jacksohn, Grösche, Rehdanz, and Schröder (2019) show that, in Germany, PV systems used to generate almost twice as much revenue for households than ST systems in 2008, but this has been reversed in the recent years.

While the information on the type of solar systems is available for all three UKLHS waves used for the analysis, it is only available for the 2015 and 2016 waves in the SOEP. For SHP, this information is not available at all. Nevertheless, I repeat the models in Table 1 with the data available, differentiating between the types of solar systems. The results, as presented in Table A4, are similar: those who live in households with solar installations are significantly more interested in politics (Models 3 and 6), irrespective of the solar system type. However, these effects disappear with the fixed-effects: if we limit the analysis to within persons, and remove any changes common to all individuals from one survey wave to another, there is no meaningful effect of solar panels on political interest (Models, 2, 3, 4, and 6).

Table A4: Effect of different types of solar systems on political attitudes

	Germany (1 - 3)			United Kingdom (4 - 6)		
	(1)	(2)	(3)	(4)	(5)	(6)
PV	0.14***	−0.01	−0.02	0.12**	0.001	0.0000
	(0.03)	(0.02)	(0.02)	(0.04)	(0.04)	(0.04)
ST	0.18***	−0.003	−0.005	0.16**	−0.08	−0.09
	(0.02)	(0.02)	(0.02)	(0.06)	(0.09)	(0.09)
PV and ST	0.16***	0.02	0.01	0.28***	−0.05	−0.06
	(0.04)	(0.04)	(0.04)	(0.06)	(0.07)	(0.07)
FEs—Individuals	No	Yes	Yes	No	Yes	Yes
FEs—Waves	No	Yes	Yes	No	Yes	Yes
Controls	No	No	Yes	No	No	Yes
Cl. SEs—Individuals	Yes	No	No	Yes	No	No
Cl. SEs—Households	Yes	Yes	Yes	Yes	Yes	Yes
Observations	257,720	257,720	239,538	103,770	103,770	102,829
R^2	0.001	0.76	0.76	0.001	0.88	0.88
Adjusted R^2	0.001	0.69	0.69	0.001	0.65	0.65

Notes: Standard errors are in parentheses. Control variables include education, head of households, unemployment, household income, households with children, house owners, and geographical regions. * $p < 0.05$, ** $p < 0.01$, *** $p < 0.001$.

Environmental Attitudes

Does solar panel ownership affect environmental attitudes? There is a mounting evidence (see the literature review by Nilsson, Bergquist, and Schultz, 2017) that engaging in one environmental behaviour can have spillover effects, negative or positive. On the one hand, producing renewable energy with their solar panels, people might feel that they are personally contributing to the fight against climate change, and therefore worry less about the environment. On the other hand, committing themselves to this fight, the cognitive dissonance theory (Festinger, 1957) would predict that they might be even more concerned than before.

I test whether solar panels affect environmental attitudes with data from the SOEP, which measures how worried the respondents are about the environment and the consequences from climate change. Both variables are measured with a three-point scale as 1 = *Very Concerned*, 2 = *Somewhat Concerned*, and 3 = *Not Concerned At All*. I have reversed coded this variable

so that the higher values indicate increasing concern, and re-run the models in Table 1 with these two concerns as the dependent variables.

Table A5 presents the regression models, returning results similar to those in the main text. The coefficient is positive and significant in the pooled model, but this significance disappears in the fixed-effect models. This suggests that those who are more concerned about the environment or climate change are more likely to adopt solar panels, but the solar panels alone do not cause a significant change in these environmental attitudes.

Table A5: Effect of solar system ownership on environmental attitudes

	Worry: Environment (1 - 3)			Worry: Climate Change (4 - 6)		
	(1)	(2)	(3)	(4)	(5)	(6)
Solar Panel	0.05***	−0.004	0.001	0.07***	−0.01	−0.01
	(0.01)	(0.01)	(0.01)	(0.01)	(0.01)	(0.01)
FEs—Individuals	No	Yes	Yes	No	Yes	Yes
FEs—Waves	No	Yes	Yes	No	Yes	Yes
Controls	No	No	Yes	No	No	Yes
Cl. SEs—Individuals	Yes	No	No	Yes	No	No
Cl. SEs—Households	Yes	Yes	Yes	Yes	Yes	Yes
Observations	273,284	273,284	254,303	234,047	234,047	217,730
R^2	0.001	0.58	0.59	0.001	0.61	0.62
Adjusted R^2	0.001	0.47	0.48	0.001	0.50	0.50

Notes: Standard errors are in parentheses. Control variables include education, head of households, unemployment, household income, households with children, house owners, and geographical regions. * $p < 0.05$, ** $p < 0.01$, *** $p < 0.001$.

Voting Intentions

Existing evidence suggests that, overall, policy feedback effects are stronger for political participation than for political attitudes (Campbell, 2012; Larsen, 2019). Accordingly, if there is any effect of solar panels on owners, we might be more likely to find it in, for example, owners' likelihood to vote rather than their interest in politics, or their likelihood to vote for green parties rather than their identification with these parties.

To test whether solar panels affect participation, I use the SHP item that measures voting intention. Specifically, it asks 'If there was an election for the National Council tomorrow, for which party would you vote?' I code *Electoral Turnout* as 0 if respondents indicated that they 'Wouldn't vote' or would vote 'For no party.' Otherwise, it is coded as 1. Similarly, I code *Vote: Greens* as 1 if they indicated the Green Party of Switzerland, Socialist Green Alternative, or the Green Liberal Party of Switzerland as their intended vote choice, and as 0 if they indicated a party other than these three parties.

Table A6 presents the regression models, returning results similar to those reported in the main text: The best estimates are substantively and statistically insignificant. In other words, solar panel ownership does not seem to have a meaningful effect on voting intentions either.

Table A6: Effect of solar system ownership on intended voting behaviour

	Electoral Turnout (1 - 3)			Vote: Greens (4 - 6)		
	(1)	(2)	(3)	(4)	(5)	(6)
Solar Panel	0.02	−0.004	−0.001	−0.001	−0.003	−0.001
	(0.02)	(0.02)	(0.02)	(0.02)	(0.03)	(0.03)
FEs—Individuals	No	Yes	Yes	No	Yes	Yes
FEs—Waves	No	Yes	Yes	No	Yes	Yes
Controls	No	No	Yes	No	No	Yes
Cl. SEs—Individuals	Yes	No	No	Yes	No	No
Cl. SEs—Households	Yes	Yes	Yes	Yes	Yes	Yes
Observations	41,800	41,800	38,905	41,800	41,800	38,905
R^2	0.0001	0.69	0.69	0.0000	0.72	0.72
Adjusted R^2	0.0000	0.57	0.57	−0.0000	0.61	0.61

Notes: Standard errors are in parentheses. Control variables include education, head of households, unemployment, household income, households with children, house owners, and geographical regions. * $p < 0.05$, ** $p < 0.01$, *** $p < 0.001$.

References

Campbell, A. L. (2012). Policy makes mass politics. *Annual Review of Political Science*, *15*, 333–351.

Festinger, L. (1957). *A theory of cognitive dissonance*. Stanford: Stanford University Press.

Jacksohn, A., Grösche, P., Rehdanz, K., and Schröder, C. (2019). Drivers of renewable technology adoption in the household sector. *Energy Economics*, *81*, 216–226.

Larsen, E. G. (2019). Policy feedback effects on mass publics: A quantitative review. *Policy Studies Journal*, *47*(2), 372–394.

Nilsson, A., Bergquist, M., and Schultz, W. P. (2017). Spillover effects in environmental behaviors, across time and context: A review and research agenda. *Environmental Education Research*, *23*(4), 573–589.
